# Presentations and outcomes of interstitial lung disease and the anti-Ro52 autoantibody

**DOI:** 10.1186/s12931-019-1231-7

**Published:** 2019-11-12

**Authors:** A. Sclafani, K. M. D’Silva, B. P. Little, E. M. Miloslavsky, J. J. Locascio, A. Sharma, S. B. Montesi

**Affiliations:** 1 Division of Pulmonary and Critical Care Medicine, Massachusetts General Hospital and Harvard Medical School, 100 Blossom St, Cox 201, Boston, MA 02114 USA; 2 Division of Rheumatology, Allergy, and Immunology, Massachusetts General Hospital and Harvard Medical School, Boston, MA USA; 3 Department of Radiology, Massachusetts General Hospital and Harvard Medical School, Boston, MA USA; 4 Biostatistics Center and Department of Neurology, Massachusetts General Hospital and Harvard Medical School, Boston, MA USA

**Keywords:** Interstitial lung disease, Myositis, Antibodies, Interstitial pneumonia with autoimmune features, Connective tissue disease

## Abstract

**Background:**

Distinct clinical presentations of interstitial lung disease (ILD) with the myositis-specific antibodies, including anti-synthetase antibodies, are well-recognized. However, the association between ILD and the myositis-associated antibodies, including anti-Ro52, is less established. Our objectives were to compare presenting phenotypes of patients with anti-Ro52 alone versus in combination with myositis-specific autoantibodies and to identify predictors of disease progression or death.

**Methods:**

We performed a retrospective cohort study of 73 adults with ILD and a positive anti-Ro52 antibody. We report clinical features, treatment, and outcomes.

**Results:**

The majority of patients with ILD and anti-Ro52 had no established connective tissue disease (78%), and one-third had no rheumatologic symptoms. Thirteen patients (17.8%) required ICU admission for respiratory failure, with 84.6% all-cause mortality. Of the 73 subjects, 85.7% had a negative SS-A, and 49.3% met criteria for idiopathic pneumonia with autoimmune features (IPAF). The 50 patients with anti-Ro52 alone were indistinguishable from patients with anti-Ro52 plus a myositis-specific autoantibody. ICU admission was associated with poor outcomes (HR 12.97, 95% CI 5.07–34.0, *p* < 0.0001), whereas rheumatologic symptoms or ANA > = 1:320 were associated with better outcomes (HR 0.4, 95% CI 0.16–0.97, *p* = 0.04, and HR 0.29, 95% CI 0.09–0.81, *p* = 0.03, respectively).

**Conclusions:**

Presentations of ILD with the anti-Ro52 antibody are heterogeneous, and outcomes are similar when compared to anti-Ro52 plus myositis-specific antibodies. Testing for anti-Ro52 may help to phenotype unclassifiable ILD patients, particularly as part of the serologic criteria for IPAF. Further research is needed to investigate treatment of ILD in the setting of anti-Ro52 positivity.

## Background

Interstitial lung disease (ILD) is a major cause of morbidity and mortality in patients with idiopathic inflammatory myopathies [1–3]. Myositis-associated ILD can present with varying degrees of respiratory compromise and muscle involvement, and may precede a diagnosis of myositis in 13 to 37.5% of cases [1, 4, 5]. Nevertheless, distinct clinical phenotypes corresponding to individual myositis autoantibodies have been identified. Even amongst the anti-synthetase antibodies (e.g. anti-Jo-1, PL-7, PL-12) classically linked with the syndrome of fever, myositis, ILD, arthritis, Raynaud’s phenomenon, and mechanic’s hands, diverse presentations are described [6–8]. Similarly, several myositis-specific autoantibodies (e.g. anti-Mi-2, TIF1gamma [p155/140], NXP-2, MDA-5) are recognized to have unique associated clinical features; anti-MDA-5, for instance, is often accompanied by skin ulceration and rapidly-progressive ILD that may be sine myositis [4, 9–12].

Less is known, however, about the presentations of ILD and many of the myositis-associated autoantibodies frequently seen in myositis overlap syndromes. One such antibody, the anti-SS-A 52 kD IgG (also known as anti-Ro52), is the most common autoantibody detected in patients with idiopathic inflammatory myopathies (estimated to be present in 20–30% of dermatomyositis/polymyositis), and has been described to co-occur with myositis-specific antibodies, particularly the anti-synthetase antibodies and anti-MDA-5 [13–18]. Importantly, the coexistence of anti-Ro52 with myositis-specific autoantibodies is associated with an aggressive ILD course [7, 19, 20].

While anti-Ro52 positivity is seen in many connective tissue diseases (CTDs), reactivity to Ro52 is greater in patients with myositis and systemic sclerosis in contrast to the anti-SS-A 60 kD antibody seen in Sjogren’s syndrome and systemic lupus erythematosus [13, 16, 21]. Although frequently conflated as simply “Ro” or “SS-A,” Ro52 and Ro60 antigens represent two different proteins, with their corresponding autoantibodies demonstrating distinct clinical associations [13, 22–24]. In one cohort of 247 patients with a high prevalence of CTD, 63% had a positive anti-Ro52 without anti-Ro60; this antibody profile was associated with pulmonary manifestations in 22% [24]. However, the diagnostic value of anti-Ro52 remains debated.

Much of the available evidence to date on the clinical associations of anti-Ro52 has been derived from patients with previously known CTD, including myositis, and from patients with positive anti-synthetase or other myositis-specific antibodies. As such, the interpretation of an isolated positive anti-Ro52 antibody is challenging as part of a diagnostic evaluation for ILD, which may be the initial manifestation of, or occur in the absence of, a specific autoimmune syndrome.

We here describe the clinical features and outcomes of 73 patients with a positive anti-Ro52 autoantibody and ILD as the presenting feature. Our study aims are 1) to characterize and describe the clinical characteristics of patients with ILD and a positive anti-Ro52, 2) to compare the presenting phenotypes of patients with anti-Ro52 alone versus in combination with a positive myositis-specific autoantibody, and 3) to identify prognostic predictors associated with ILD progression or death.

## Methods

### Study design, setting, and participants

We performed a retrospective cohort study of all adult patients seen at Massachusetts General Hospital with ILD and a positive anti-Ro52 antibody from January 1, 2015 to August 16, 2018. Patients were identified from a centralized Research Patient Data Registry if they met the following criteria: 1) age greater than 18 years, 2) diagnosis of ILD according to International Classification of Diseases (10th Revision) code J84 (other interstitial pulmonary diseases), and 3) presence of a positive anti-Ro52 (positive values were indicated by a titer of ≧20 units on one standard enzyme immunoassay, the MyoMarker Panel 3, RDL Reference Laboratory^1^). Clinical charts of identified patients were reviewed to ensure inclusion criteria were met and the diagnosis of ILD confirmed on imaging. We excluded a total of 5 patients without chest computed tomography (CT) or whose CTs were not consistent with ILD on review by subspecialty thoracic radiologists. This study was approved by the Partners Institutional Review Board.

### Data collection

Clinical data were systematically collected for each patient by one author (A.Sc.) through a review of the electronic medical record. Clinical variables of interest were recorded at the time of anti-Ro52 positivity, including demographics, duration of pulmonary symptoms, intensive care unit (ICU) admission, rheumatologic symptoms, and prior CTD diagnosis. Laboratory results including anti-Ro52 titer by enzyme immunoassay, antinuclear antibody (ANA), SS-A by enzyme-linked immunosorbent assay (ELISA), creatine kinase, aldolase, and myositis-specific autoantibodies including the anti-synthetase antibodies were also collected. When available, lung biopsy histopathology, pulmonary function testing (PFTs), and immunosuppressive or antifibrotic treatments received after anti-Ro52 testing were recorded.

CTs closest to the time of anti-Ro52 positivity were reviewed independently by two thoracic radiologists (A.Sh. and B.P.L.) and categorized by consensus into ILD patterns according to the ATS/ERS classification of the Idiopathic Interstitial Pneumonias [25]. Presence of fibrosis as defined by reticulation, traction bronchiectasis, and/or honeycombing was reported. Two rheumatologists (E.M.M. and K.D.) independently reviewed each medical record and determined by consensus whether patients met accepted society guideline criteria for CTD at the time of anti-Ro52 testing [8, 26–32]. Additionally, patients were classified as meeting criteria for interstitial pneumonia with autoimmune features (IPAF) according to the 2015 ERS/ATS task force research statement [33].

Outcomes were reported at the time of most recent available follow-up for each patient, which was variable and included both inpatient and outpatient evaluations. Improvement was defined by a greater than 10% increase in forced vital capacity (FVC) on PFTs or clinical improvement (decreased symptoms, improved oxygenation, or hospital discharge) if PFTs were not available, stability by a less than 10% change in FVC or clinical stability if PFTs were not available, and progression by a greater than 10% decline in FVC or clinical worsening if PFTs were not available. As a primary respiratory cause of death was not able to be identified in many patients with multiorgan failure at the time of death, mortality was reported as all-cause.

### Statistical analysis

Continuous variables are presented as medians with ranges, and categorical variables are summarized as counts and percentages. All statistical tests (Wilcoxon rank-sum test for continuous variables and Chi-squared or Fisher’s exact tests for categorical variables) were two-sided. *P* values less than 0.05 were considered to indicate statistical significance. Corrections for multiple comparisons were applied using the step-down Sidak method. Cox proportional hazards regression was performed predicting time (follow-up days) to poor outcome (disease progression or death). Patients who were stable or improved were censored. A backward elimination algorithm was applied to the predictors age, gender, ICU admission, presence of any rheumatologic symptom, ANA positivity, anti-Ro52 titer, presence of myositis-specific antibodies, fibrosis on imaging, and IPAF criteria met. Statistical analyses were performed using Stata 15.1 (StataCorp) and SAS 9.4 (SAS Institute Inc).

## Results

### Clinical features of anti-Ro52 patients

A total of 73 patients were identified with ILD and a positive anti-Ro52 antibody between January 2015 (when Ro52 testing first became available at our institution) and August 2018. Ages ranged from 23 to 90 years old, with median 68 (Table 1). Thirteen patients (17.8%) required ICU admission for respiratory failure at the time of antibody testing. One-third of patients had no rheumatologic symptoms on presentation, and nearly one-third experienced myalgias. The majority (78%) did not carry a prior CTD diagnosis, and only 27.4% met CTD diagnostic criteria after anti-Ro52 testing. Anti-synthetase syndrome and dermatomyositis/polymyositis were diagnosed in 5 patients (6.9%) each. 36 patients (49.3%) met IPAF criteria.

**Table 1:** Demographics and clinical features of patients with ILD and a positive anti-Ro52 antibody. Clinical features were compared between patients with anti-Ro52 alone vs anti-Ro52 plus an additional myositis-specific autoantibody (including anti-Jo-1, anti-PL-12, anti-PL-7, anti-EJ, anti-OJ, anti-Mi-2, anti-SRP, anti-MDA5, anti-NXP2, or anti-TIF-1γ)

Variable	All patients *n* = 73	Stratified by Autoantibodies			
		Isolated Anti-Ro52 *n* = 50	Anti-Ro52 plus myositis-specific autoAb *n* = 23	Raw *p*-value	Adjusted *p*-value*
Age, years median [range]	68 [23–90]	68.5 [23–90]	68 [37–83]	0.75	1.0
Male, n (%)	45 (61.6%)	31 (62%)	14 (60.9%)	0.93	1.0
Ethnicity, n (%)				0.53	1.0
African American	7 (9.6%)	5 (10%)	2 (8.7%)		
Asian	6 (8.2%)	3 (6.0%)	3 (13%)		
Hispanic	4 (5.5%)	2 (4.0%)	2 (8.7%)		
White	56 (76.7%)	40 (80%)	16 (69.6%)		
Smoking history, n (%)				0.43	1.0
Current/Former	43 (58.9%)	31 (62%)	12 (52.2%)		
Never	30 (41.1%)	19 (38%)	11 (47.8%)		
Duration of pulmonary symptoms, n (%)				0.59	1.0
Acute (<1 week)	9 (12.3%)	7 (14%)	2 (8.7%)		
Subacute (1 week-6 mo)	14 (19.2%)	8 (16%)	6 (26.1%)		
Chronic (>6 months)	48 (65.8%)	34 (68%)	14 (60.9%)		
None	2 (2.7%)	1 (2%)	1 (4.4%)		
ICU admission on presentation, n (%)	13 (17.8%)	10 (20%)	3 (13%)	0.74	1.0
Rheumatologic symptoms, n (%)					
Myalgias	23 (31.5%)	15 (30%)	8 (34.8%)	0.68	1.0
Mechanic’s hands	10 (13.7%)	3 (6%)	7 (30.4%)	0.01	0.24
Gottron’s papules	2 (2.7%)	1 (2%)	1 (4.4%)	0.53	1.0
Heliotrope rash	2 (2.7%)	2 (4%)	0 (0%)	1.0	1.0
Other rash	13 (17.8%)	7 (14%)	6 (26.1%)	0.32	1.0
Weight loss	19 (26%)	13 (26%)	6 (26.1%)	0.99	1.0
Raynaud’s	18 (24.7%)	12 (24%)	6 (26.1%)	0.85	1.0
Arthralgias	16 (21.9%)	10 (20%)	6 (26.1%)	0.56	1.0
Sicca symptoms	13 (17.8%)	9 (18%)	4 (17.4%)	1.0	1.0
Alopecia	5 (6.8%)	4 (8%)	1 (4.4%)	1.0	1.0
Scleroderma/dactyly	7 (9.6%)	6 (12%)	1 (4.4%)	0.42	1.0
Any rheum symptoms	49 (67.1%)	35 (70%)	14 (60.9%)	0.44	1.0
None	24 (32.9%)	15 (30%)	9 (39.1%)	0.44	1.0
Prior CTD diagnosis, n (%)				0.97	1.0
RA	1 (1.4%)	1 (2%)	0 (0%)		
Sjogren’s	3 (4.1%)	2 (4%)	1 (4.4%)		
Scleroderma	4 (5.5%)	3 (6%)	1 (4.4%)		
SLE	3 (4.1%)	2 (4%)	1 (4.4%)		
DM/PM	2 (2.7%)	1 (2%)	1 (3.7%)		
MCTD	3 (4.1%)	3 (6%)	0 (0%)		
None	57 (78%)	38 (76%)	19 (82.6%)		
Met diagnostic criteria for a CTD, n (%)				0.02	0.48
Anti-synthetase	5 (6.9%)	0 (0%)	5 (21.7%)		
DM/PM	5 (6.9%)	3 (6%)	2 (8.7%)		
MCTD	3 (4.1%)	3 (6%)	0 (0%)		
PMR	1 (1.4%)	1 (2%)	0 (0%)		
SLE	2 (2.7%)	1 (2%)	1 (4.4%)		
Scleroderma	4 (5.5%)	3 (6%)	1 (4.4%)		
None	53 (72.6%)	39 (78%)	14 (60.9%)		
Met IPAF criteria, n (%)	36 (49.3%)	27 (54%)	9 (39.1%)	0.24	1.0

*Corrected for multiple comparisons using the step-down Sidak method

ANA was negative in 17.8% of patients and positive at a titer ≥1:320 in 39.7% (Table 2). Most patients (85.7%) were SS-A negative by ELISA. The most common radiographic patterns were nonspecific interstitial pneumonia (NSIP), organizing pneumonia (OP), mixed NSIP/OP, and usual interstitial pneumonia (UIP), with half of patients having CT evidence of fibrosis. Corticosteroids were the most common pharmacotherapy (given to all patients admitted to the ICU and more than half of non-ICU patients), with half of patients receiving rituximab or mycophenolate either in combination with steroids or as monotherapy (Table 3).

**Table 2:** Laboratory profile, imaging, and histopathology of patients with ILD and a positive anti-Ro52 antibody

Variable	All patients *n* = 73	Stratified by Autoantibodies			
		Isolated Anti-Ro52 *n* = 50	Anti-Ro52 plus myositis-specific autoAb *n* = 23	Raw *p*-value	Adjusted *p*-value*
Anti-Ro52 titer (nl < 20 Units), median [interquartile range]	59 [31–122]	44.5 [28–98]	108 [41–140]	0.01	0.25
ANA, n (%)				0.07	0.85
Negative	13 (17.8%)	9 (18%)	4 (17.4%)		
Positive titer <1:320	31 (42.5%)	17 (34%)	14 (60.9%)		
Positive titer > = 1:320	29 (39.7%)	24 (48%)	5 (21.7%)		
Creatine kinase (nl < 400 U/L), median [range]	110 [13–13,965] (*n* = 63)	102 [13–13,965] (*n* = 42)	119 [21–1774] (*n* = 21)	0.73	1.0
Aldolase (nl < 7.7 U/L), median [range]	7.90 [3.8–273] (*n* = 67)	8.0 [3.9–273] (*n* = 45)	7.15 [3.8–45] (*n* = 22)	0.25	1.0
SS-A ELISA positive, n (%)	9 (14.3%) (*n* = 63)	8 (18.2%) (*n* = 44)	1 (5.3%) (*n* = 19)	0.26	1.0
Positive cytoplasmic Ab, n (%)	17 (23.3%)	5 (10%)	12 (52.2%)	<0.0001	0.002
Dominant radiographic pattern, n (%)				0.93	1.0
OP	25 (34.3%)	16 (32%)	9 (39.1%)		
NSIP	14 (19.2%)	10 (20%)	4 (17.4%)		
UIP	13 (17.8%)	8 (16%)	5 (21.7%)		
Mixed OP/NSIP	10 (13.7%)	8 (16%)	2 (8.7%)		
HP	4 (5.5%)	3 (6%)	1 (4.4%)		
Sarcoid	2 (2.7%)	1 (2%)	1 (4.4%)		
LIP	1 (1.4%)	1 (2%)	0 (0%)		
DAD	1 (1.4%)	1 (2%)	0 (0%)		
PPFE	1 (1.4%)	1 (2%)	0 (0%)		
Infection/aspiration	1 (1.4%)	1 (2%)	0 (0%)		
Other	1 (1.4%)	0 (0%)	1 (4.4%)		
Fibrosis present, n (%)	37 (50.7%)	25 (50%)	12 (52.2%)	0.86	1.0
Lung biopsy pathology, n (%)				0.07	0.85
OP	7 (9.6%)	5 (10%)	2 (8.7%)		
NSIP	3 (4.1%)	3 (6%)	0 (0%)		
UIP	1 (1.4%)	0 (0%)	1 (4.4%)		
Mixed OP/NSIP	3 (4.1%)	0 (0%)	3 (13%)		
HP	1 (1.4%)	1 (2%)	0 (0%)		
Sarcoid	1 (1.4%)	1 (2%)	0 (0%)		
No biopsy	57 (78.1%)	40 (80%)	17 (73.9%)		

*Corrected for multiple comparisons using the step-down Sidak method

**Table 3:** Treatment and outcomes of patients with ILD and a positive anti-Ro52 antibody, stratified by ICU vs non-ICU (e.g. hospital or clinic) presentation at the time of anti-Ro52 lab testing. Pharmacotherapy includes all medications given, including in combination. Antifibrotic therapy includes medications approved for the treatment of idiopathic pulmonary fibrosis, including pirfenidone or nintedanib. Mortality is all-cause

Variable	All patients *n* = 73	ICU presentation *n* = 13	Non-ICU presentation *n* = 60
Age, years median [range]	68 [23–90]	60 [43–86]	69 [23–90]
Male, n (%)	45 (61.6%)	10 (76.9%)	35 (58.3%)
Ethnicity, n (%)			
African American	7 (9.6%)	2 (15.4%)	5 (8.3%)
Asian	6 (8.2%)	2 (15.4%)	4 (6.7%)
Hispanic	4 (5.5%)	0 (0%)	4 (6.7%)
White	56 (76.7%)	9 (69.2%)	47 (78.3%)
Smoking history, n (%)			
Current/Former	43 (58.9%)	10 (76.9%)	33 (55%)
Never	30 (41.1%)	3 (23.1%)	27 (45%)
Pharmacotherapy, n (%)			
Corticosteroids	45 (61.6%)	13 (100%)	32 (53.3%)
Rituximab	19 (26.0%)	2 (15.4%)	17 (28.3%)
IVIG	8 (11%)	4 (30.8%)	4 (6.7%)
Mycophenolate	18 (24.7%)	2 (15.4%)	16 (26.7%)
Cyclophosphamide	2 (2.7%)	1 (7.7%)	1 (1.7%)
Antifibrotic	5 (6.9%)	0 (0%)	5 (8.3%)
Outcome, n (%)			
Improved	12 (16.4%)	2 (15.4%)	10 (16.7%)
Stable	32 (43.8%)		32 (53.3%)
Progressed	8 (11%)		8 (13.3%)
Died	21 (28.8%)	11 (84.6%)	10 (16.7%)

### Isolated anti-Ro52 vs anti-Ro52 plus an additional myositis-specific autoantibody

Fifty of 73 patients (68%) had an isolated positive anti-Ro52 (i.e. in the absence of a myositis-specific antibody), and 23 additionally had at least one positive myositis-specific autoantibody, including PL-7 (7 patients), Jo-1 (6 patients), and MDA-5 (5 patients). Other positive antibodies present in two or fewer patients included PL-12, Mi-2, TIF1gamma (p155/140), and NXP-2 (Tables 1 and 2). Although there was a trend towards greater prevalence of mechanic’s hands, meeting diagnostic criteria for CTD, and higher anti-Ro52 titer in patients with an additional myositis-specific antibody, after correcting for multiple comparisons only a positive cytoplasmic antibody was statistically significantly associated with the presence of an additional positive myositis-specific antibody compared to anti-Ro52 alone (*p* = 0.002).

### Predictors of poor outcome

**Fig. 1:**
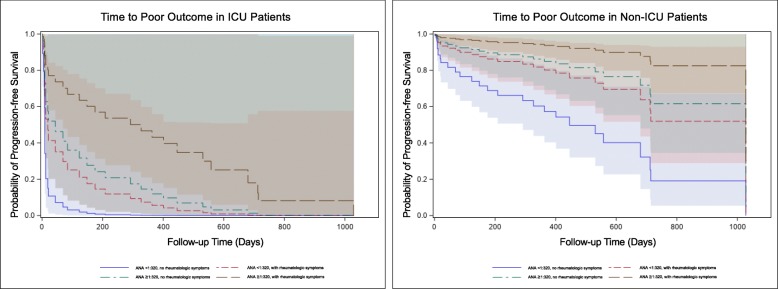
Kaplan-Meier curves indicating estimated proportion of patients not yet showing a poor outcome (defined as a composite of disease progression or death) versus follow-up days, stratified by ICU admission at the time of anti-Ro52 antibody testing. Admission to an ICU was associated with a higher risk of poor outcome (HR 12.97, 95% CI 5.07–34.0, *p* < 0.0001), whereas presence of any rheumatologic symptom or high titer ANA > = 1:320 were associated with a lower risk of poor outcome (HR 0.4, 95% CI 0.16–0.97, *p* = 0.04, and HR 0.29, 95% CI 0.09–0.81, *p* = 0.03, respectively)

All-cause mortality was 28.8%. Mortality was largely driven by the 84.6% mortality of patients admitted to the ICU with respiratory failure at the time of anti-Ro52 testing (Table 3). There was no unadjusted difference in outcomes (disease progression or death) in patients with isolated anti-Ro52 versus with an additional myositis-specific antibody (36% vs 47.8%, *p* = 0.34). In Cox proportional hazards regression analysis, admission to an ICU was associated with a higher risk of poor outcome (HR 12.97, 95% CI 5.07–34.0, *p* < 0.0001), whereas presence of any rheumatologic symptom or high titer ANA > = 1:320 were both associated with a lower risk of poor outcome (HR 0.4, 95% CI 0.16–0.97, *p* = 0.04, and HR 0.29, 95% CI 0.09–0.81, *p* = 0.03, respectively) (Fig. 1). There was no statistically significant association between poor outcome and radiographic pattern, imaging evidence of fibrosis, or anti-Ro52 titer.

## Discussion

We present the results of the largest, to our knowledge, described cohort of patients with the anti-Ro52 antibody and ILD. While the association of ILD with the myositis-specific antibodies is well-recognized, the association between ILD and the myositis-associated antibodies, including anti-Ro52, is less established. We found that clinical presentations of ILD patients with a positive anti-Ro52 antibody were heterogeneous, ranging from the absence of pulmonary symptoms to hypoxemic respiratory failure with a wide range of ages, men and women, and smokers and non-smokers affected. In our cohort, patients with isolated anti-Ro52 were indistinguishable in presentation and outcomes from patients with anti-Ro52 in combination with myositis-specific autoantibodies. Our results suggest that the presence of a positive anti-Ro52 may have important implications for patients with ILD similar to the presence of a positive myositis-specific antibody.

Prior case series have described an aggressive phenotype of ILD, including worse disease severity and rapid progression, in patients with anti-synthetase antibodies co-existing with anti-Ro52 [7, 19]. Our findings suggest that anti-Ro52 alone is associated with similar outcomes when compared to anti-Ro52 in combination with myositis-specific antibodies. Similar to other reports, the imaging and histopathologic patterns of NSIP and organizing pneumonia seen in our cohort reflect recognized patterns associated with ILD in the idiopathic inflammatory myopathies and other CTDs [1, 8, 34]. Additionally, we found that the presence of positive cytoplasmic antibodies in the myositis-specific group was the only statistically significant difference between these patients and those with anti-Ro52 alone. Cytoplasmic staining on immunofluorescence found during ANA testing has been described in anti-synthetase syndrome and should alert clinicians to test for myositis antibodies particularly in the context of ILD [9, 35].

Published evidence suggests that as much as 20% of anti-Ro52 or Ro60 reactivity can be missed when testing for this with many commercially-available SS-A ELISAs that are based on a mixture of both the 60 kD and 52 kD antigens [22]. The observation that diagnostic assays using blended antigens may not detect single reactivity to either antigen has led to recommendations to test for each separately [22–24]. As the vast majority (more than 85%) of patients in our cohort were anti-Ro52 positive by enzyme immunoassay but SS-A/Ro negative by ELISA, our data confirms that many Ro52 positive patients may be missed if SS-A via combination ELISA is used for screening. This is especially relevant given that “anti-Ro (SS-A)” is part of the serologic domain for interstitial pneumonia with autoimmune features [33]. This classification is increasingly used in research and clinical practice for undifferentiated ILD [36, 37]. Deliberate anti-Ro52 testing may therefore help to phenotype patients with previously unclassifiable ILD. In our cohort only one-fourth of patients met criteria for CTD. Testing for SS-A via ELISA alone would have classified only 30.1% of patients as having IPAF; thus, specific testing for anti-Ro52 resulted in a net reclassification of 14 patients (19.2%), thereby increasing the total number of IPAF patients in this cohort to 36 (49.3%).

We observed a strikingly high mortality, nearly 85%, in the patients that tested positive for anti-Ro52 while in the ICU, suggesting that this is an important group of patients to identify. This mortality rate is more than double that of acute respiratory distress syndrome [38], and closer to that of respiratory failure in idiopathic pulmonary fibrosis than in CTD-associated ILD [39]. Potential explanations for this high mortality are only speculative. We reported all-cause mortality and as such our results may overestimate the number of respiratory-related deaths. While it is not surprising that ICU admission was associated with worse outcome, after controlling for this and other clinically relevant predictors we also found that both presence of rheumatologic symptoms and positive ANA > = 1:320 were associated with a lower risk of disease progression or death. Given the small numbers of patients in each treatment category our study was underpowered to investigate whether this was due to any treatment effect, including whether such patients were more likely to receive immunosuppression, and this should be investigated in future studies.

Our study has several important limitations. First, this is a retrospective, single center study with small sample size and variable patient follow-up intervals. A future prospective study to confirm our findings is warranted. Second, we cannot prove causality between the presence of an anti-Ro52 antibody and the presence of ILD since a pathophysiologic mechanism remains unknown; however, this is also true of the anti-synthetase antibodies that are generally accepted to be associated with ILD. Third, there are varying estimates of the prevalence of anti-Ro52 in healthy individuals, with the highest estimate approximately 12% based on a study of 100 subjects [40], and we are unable to provide a true estimate of the prevalence of anti-Ro52 in patients with ILD to determine how this compares to that found in healthy controls. Finally, we were unable to comment on the effect of treatment on outcomes due to our small sample size. Further research is needed to assess the prognostic and treatment implications of ILD in in the setting of a positive anti-Ro52 autoantibody.

## Conclusions

Presentations of ILD with a positive anti-Ro52 antibody are heterogeneous and occur frequently in the absence of rheumatologic symptoms or prior diagnosis of CTD. In our cohort, clinical features and outcomes of ILD with an isolated anti-Ro52 were similar to ILD with anti-Ro52 in combination with myositis-specific antibodies, including the anti-synthetase antibodies. Mortality is high, especially in the context of ICU admission. Use of the SS-A ELISA alone as the diagnostic test for anti-Ro52 misses a large number of anti-Ro52 positive patients. Dedicated testing for anti-Ro52 may help to phenotype unclassifiable ILD patients, particularly as part of the serologic criteria for IPAF, which may have important prognostic and future therapeutic implications. Further research is needed to assess treatment strategies for ILD in the setting of anti-Ro52 positivity.

## Data Availability

The datasets used and/or analyzed during the current study are available from the corresponding author on reasonable request.

## Abbreviations

ANA: Antinuclear antibody
CK: Creatine kinase
CT: Computed tomography
CTD: Connect tissue disease
DAD: Diffuse alveolar damage
DM/PM: Dermatomyositis/polymyositis
FVC: Forced vital capacity
HP: Hypersensitivity pneumonitis
ILD: Interstitial lung disease
IPAF: Idiopathic pneumonia with autoimmune features
IVIG: Intravenous immune globulin
LIP: Lymphocytic interstitial pneumonia
MCTD: Mixed connective tissue disease
NSIP: Non-specific interstitial pneumonia
OP: Organizing pneumonia
PFT: Pulmonary function test
PPFE: Pleuroparenchymal fibroelastosis
RA: Rheumatoid arthritis
SLE: Systemic lupus erythematosus
UIP: Usual interstitial pneumonia

## Footnotes

1 MyoMarker Panel 3 (RDL Reference Laboratory) includes the following tests: Anti-Jo-1 Ab, Anti-Mi-2 Ab, Anti-PL-12 Ab, Anti-PL-7 Ab, Anti-EJ Ab, Anti-OJ Ab, Anti-SRP Ab, Anti-Ku Ab, Anti-U2 RNP, Anti-PM/Scl-100 Ab, Anti-MDA5 Ab, Anti-NXP2 Ab, Anti-TIF-1γ Ab, Anti-SSA 52 kD IgG Ab, Anti-U1 RNP Ab, Anti-Fibrillarin U3 RNP Ab. http://www.rdlinc.com/test_menu/myomarker-panel-3/
